# Efficacy and effectiveness of an rVSV-vectored vaccine in preventing Ebola virus disease: final results from the Guinea ring vaccination, open-label, cluster-randomised trial (Ebola Ça Suffit!)

**DOI:** 10.1016/S0140-6736(16)32621-6

**Published:** 2017-02-04

**Authors:** Ana Maria Henao-Restrepo, Anton Camacho, Ira M Longini, Conall H Watson, W John Edmunds, Matthias Egger, Miles W Carroll, Natalie E Dean, Ibrahima Diatta, Moussa Doumbia, Bertrand Draguez, Sophie Duraffour, Godwin Enwere, Rebecca Grais, Stephan Gunther, Pierre-Stéphane Gsell, Stefanie Hossmann, Sara Viksmoen Watle, Mandy Kader Kondé, Sakoba Kéïta, Souleymane Kone, Eewa Kuisma, Myron M Levine, Sema Mandal, Thomas Mauget, Gunnstein Norheim, Ximena Riveros, Aboubacar Soumah, Sven Trelle, Andrea S Vicari, John-Arne Røttingen, Marie-Paule Kieny

**Affiliations:** a WHO, Geneva, Switzerland; b Faculty of Epidemiology and Population Health, London School of Hygiene & Tropical Medicine, London, UK; c Department of Biostatistics, University of Florida, Gainesville, FL, USA; d Institute of Social and Preventive Medicine, University of Bern, Bern, Switzerland; e Centre for Infectious Disease Epidemiology and Research, University of Cape Town, Cape Town, South Africa; f Public Health England, London, UK; g Centre National d'Appui à la Lutte contre la Maladie, Bamako, Mali; h Médecins Sans Frontières, Brussels, Belgium; i Bernard Nocht Institute for Tropical Medicine, University of Hamburg, Hamburg, Germany; j Epicentre, Paris, France; k Clinical Trials Unit Bern, University of Bern, Bern, Switzerland; l Center Of Excellence For Training, Research On Malaria & Priority Diseases In Guinea, Conakry, Guinea; m Ebola Response, Ministry of Health, Conakry, Guinea; n Center for Vaccine Development, University of Maryland School of Medicine, Baltimore, MD, USA; o Division of Infectious Disease Control, Norwegian Institute of Public Health, Oslo, Norway; p Department of Health and Society, University of Oslo, Norway; q Department of Global Health and Population, Harvard TH Chan School of Public Health, Boston, MA, USA; r Coalition for Epidemic Preparedness Innovations, care of Norwegian Institute of Public Health, Oslo, Norway

## Abstract

**Background:**

rVSV-ZEBOV is a recombinant, replication competent vesicular stomatitis virus-based candidate vaccine expressing a surface glycoprotein of Zaire Ebolavirus. We tested the effect of rVSV-ZEBOV in preventing Ebola virus disease in contacts and contacts of contacts of recently confirmed cases in Guinea, west Africa.

**Methods:**

We did an open-label, cluster-randomised ring vaccination trial (Ebola ça Suffit!) in the communities of Conakry and eight surrounding prefectures in the Basse-Guinée region of Guinea, and in Tomkolili and Bombali in Sierra Leone. We assessed the efficacy of a single intramuscular dose of rVSV-ZEBOV (2×10^7^ plaque-forming units administered in the deltoid muscle) in the prevention of laboratory confirmed Ebola virus disease. After confirmation of a case of Ebola virus disease, we definitively enumerated on a list a ring (cluster) of all their contacts and contacts of contacts including named contacts and contacts of contacts who were absent at the time of the trial team visit. The list was archived, then we randomly assigned clusters (1:1) to either immediate vaccination or delayed vaccination (21 days later) of all eligible individuals (eg, those aged ≥18 years and not pregnant, breastfeeding, or severely ill). An independent statistician generated the assignment sequence using block randomisation with randomly varying blocks, stratified by location (urban *vs* rural) and size of rings (≤20 individuals *vs* >20 individuals). Ebola response teams and laboratory workers were unaware of assignments. After a recommendation by an independent data and safety monitoring board, randomisation was stopped and immediate vaccination was also offered to children aged 6–17 years and all identified rings. The prespecified primary outcome was a laboratory confirmed case of Ebola virus disease with onset 10 days or more from randomisation. The primary analysis compared the incidence of Ebola virus disease in eligible and vaccinated individuals assigned to immediate vaccination versus eligible contacts and contacts of contacts assigned to delayed vaccination. This trial is registered with the Pan African Clinical Trials Registry, number PACTR201503001057193.

**Findings:**

In the randomised part of the trial we identified 4539 contacts and contacts of contacts in 51 clusters randomly assigned to immediate vaccination (of whom 3232 were eligible, 2151 consented, and 2119 were immediately vaccinated) and 4557 contacts and contacts of contacts in 47 clusters randomly assigned to delayed vaccination (of whom 3096 were eligible, 2539 consented, and 2041 were vaccinated 21 days after randomisation). No cases of Ebola virus disease occurred 10 days or more after randomisation among randomly assigned contacts and contacts of contacts vaccinated in immediate clusters versus 16 cases (7 clusters affected) among all eligible individuals in delayed clusters. Vaccine efficacy was 100% (95% CI 68·9–100·0, p=0·0045), and the calculated intraclass correlation coefficient was 0·035. Additionally, we defined 19 non-randomised clusters in which we enumerated 2745 contacts and contacts of contacts, 2006 of whom were eligible and 1677 were immediately vaccinated, including 194 children. The evidence from all 117 clusters showed that no cases of Ebola virus disease occurred 10 days or more after randomisation among all immediately vaccinated contacts and contacts of contacts versus 23 cases (11 clusters affected) among all eligible contacts and contacts of contacts in delayed plus all eligible contacts and contacts of contacts never vaccinated in immediate clusters. The estimated vaccine efficacy here was 100% (95% CI 79·3–100·0, p=0·0033). 52% of contacts and contacts of contacts assigned to immediate vaccination and in non-randomised clusters received the vaccine immediately; vaccination protected both vaccinated and unvaccinated people in those clusters. 5837 individuals in total received the vaccine (5643 adults and 194 children), and all vaccinees were followed up for 84 days. 3149 (53·9%) of 5837 individuals reported at least one adverse event in the 14 days after vaccination; these were typically mild (87·5% of all 7211 adverse events). Headache (1832 [25·4%]), fatigue (1361 [18·9%]), and muscle pain (942 [13·1%]) were the most commonly reported adverse events in this period across all age groups. 80 serious adverse events were identified, of which two were judged to be related to vaccination (one febrile reaction and one anaphylaxis) and one possibly related (influenza-like illness); all three recovered without sequelae.

**Interpretation:**

The results add weight to the interim assessment that rVSV-ZEBOV offers substantial protection against Ebola virus disease, with no cases among vaccinated individuals from day 10 after vaccination in both randomised and non-randomised clusters.

**Funding:**

WHO, UK Wellcome Trust, the UK Government through the Department of International Development, Médecins Sans Frontières, Norwegian Ministry of Foreign Affairs (through the Research Council of Norway's GLOBVAC programme), and the Canadian Government (through the Public Health Agency of Canada, Canadian Institutes of Health Research, International Development Research Centre and Department of Foreign Affairs, Trade and Development).

## Introduction

Since the Ebola virus was first identified in 1976, sporadic outbreaks of Ebola virus disease have been reported in Africa, each causing high mortality.^1^ No vaccine is currently licensed for preventing Ebola virus disease or other filovirus infections. The 2013–16 outbreak of Ebola virus disease in west Africa^2^ highlighted the need to produce and assess a safe and effective Ebola vaccine for human beings.^3^ One promising vaccine candidate,^4^ the recombinant, replication-competent, vesicular stomatitis virus-based vaccine expressing the glycoprotein of a Zaire Ebolavirus (rVSV-ZEBOV), is protective in challenge models in several animal species,^5^, ^6^, ^7^, ^8^, ^9^, ^10^, ^11^, ^12^, ^13^, ^14^, ^15^, ^16^ including mice, hamsters, guinea pigs, and non-human primates.^4^, ^5^ A single dose completely protected non-human primates against high-dose challenge (around 1000 particle-forming units) when administered between 7 and 31 days pre-challenge^7^, ^8^, ^9^ and partly protected non-human primates when administered from 3 days before^7^ to 24 h after challenge with the Makona strain responsible for the west African epidemic.^11^

We therefore undertook Ebola ça Suffit! (translated as “Ebola that's enough!”), a ring vaccination phase 3 efficacy trial in Guinea whose primary objective was to assess the efficacy of the rVSV-ZEBOV vaccine for the prevention of Ebola virus disease in human beings (the ring vaccination approach was inspired by the surveillance-containment strategy that led to smallpox eradication).^17^ Preliminary results indicated 100% vaccine efficacy (95% CI 74·7–100·0) at interim analysis, after which the delayed-vaccination arm was discontinued.^18^ Here, we present the final results of the trial.

**Research in context**

**Evidence before this study**

There are currently no licensed vaccines for preventing Ebola virus disease or other filovirus infections. The rVSV-ZEBOV candidate vaccine has been reported to be protective in challenge models in several non-human species. We searched Medline and EMBASE without language restrictions for articles published from January, 1990, to July 20, 2015, to identify any published phase 3 clinical trials assessing the efficacy of Ebola vaccines, using the search terms “Ebola virus”, “filovirus”, “prophylaxis”, “vaccine”, and “clinical trials”. The rVSV-ZEBOV vaccine has been studied in phase 1 and phase 2 studies, which have documented its immunogenicity and safety profile. To our knowledge, ours is the only phase 3 trial of this vaccine in west Africa that has reported results, and no trial until now has used the ring vaccination cluster-randomised design. Therefore, we could not do a detailed systematic review at this point in time.

**Added value of this study**

Ebola Ça Suffit used a novel trial design based on identification of people at risk around a newly confirmed case of Ebola virus disease (contacts and contacts of contacts) and ring vaccination to improve the prospect of generating robust evidence on the effects of the vaccine despite the low and decreasing incidence of Ebola virus disease. Individuals were either randomly assigned to immediate vaccination or delayed vaccination, or not randomly assigned (and received immediate vaccination). Interim analysis suggested that rVSV-ZEBOV offered very high protection, leading to the delayed-vaccination arm being discontinued. Final data from all trial clusters (randomised and non-randomised, with children included in the non-randomised group) showed that at 10 days or more after randomisation, there were no cases of Ebola virus disease among immediately vaccinated contacts and contacts of contacts; ie, 100% protection. Adverse events data indicated no safety concerns in adults or children.

**Implications of all the available evidence**

We used a novel trial design, which had a high probability of generating evidence on the individual and cluster-level effects of the vaccine despite the low and decreasing incidence of Ebola virus disease. These results indicate that rVSV-ZEBOV is safe and effective in averting Ebola virus disease when added to established control measures as a ring vaccination approach. Ring vaccination trials might have application in the assessment of other vaccine candidates in epidemics of other viral haemorrhagic fevers or other emerging infectious diseases.

## Methods

### Study design and participants

The Guinea ring vaccination trial was a cluster-randomised controlled trial designed to assess the effect of one dose of the candidate vaccine in protecting against laboratory confirmed Ebola virus disease. We did this trial in the community in Conakry and eight surrounding prefectures in the Basse-Guinée region of Guinea (appendix).

The Guinean national medicines regulatory agency (Direction Nationale de la Pharmacie et du Laboratoire) and the national ethics committee (Comité National d'Ethique pour la Recherche en Santé), the WHO Ethical Research Committee, and Norwegian Regional Committees for Medical and Health Research Ethics approved the study protocol. In Aug, 2015, after approval by Sierra Leonean National Regulatory Authority and the Ethics Review Committee, the trial was extended to Sierra Leone (Tomkolili and Bombali).

Ebola virus spread across many geographical areas of Guinea, mainly through familial and social networks and funeral exposures.^19^ After confirmation of a case of Ebola virus disease (index case), we enumerated and randomised clusters (called rings) of epidemiologically linked people.^20^ The ring vaccination design ensured that the study was undertaken in pockets of high incidence of Ebola virus disease despite the declining epidemic and an overall low attack rate (ie, the total number of cases of Ebola virus disease in the three worst affected countries divided by the estimated total population of these countries; estimated here as about 0·13%). Details of the study protocol, study team composition, study procedures, and statistical analysis plan have been previously reported.^18^, ^20^

Briefly, we enumerated clusters as a list of all contacts and contacts of contacts of the index case including residents temporarily absent at the time of enumeration. We defined contacts as individuals who lived in the same household, visited or were visited by the index case after the onset of symptoms, provided him or her with unprotected care, or prepared the body for the traditional funeral ceremony. These contacts included high-risk contacts who were in close physical contact with the patient's body or body fluids, linen, or clothes.^21^ Contacts of contacts were the neighbours of the index case to the nearest appropriate geographical boundary plus the household members of any high-risk contacts living away from the index cases' residence. A new cluster was defined if at least 60% of the contacts and contacts of contacts were not enumerated in a previous cluster.

We randomly assigned clusters into immediate vaccination or vaccination delayed by 21 days. Exclusion criteria were: history of Ebola virus disease (self-declared or laboratory confirmed), being aged less than 18 years, pregnancy (verbally declared) or breastfeeding (women were invited, but not forced, to take a pregnancy test), history of administration of other experimental treatments during the past 28 days, history of anaphylaxis to a vaccine or vaccine component, or serious disease requiring confining to bed or admission to hospital by the time of vaccination. Within each cluster, all people who were eligible and consented were offered vaccination.

A team obtained written informed consent from all eligible contacts and contacts of contacts using a printed information sheet. If the person in question was illiterate, these documents were read to him or her in their local language and a fingerprint from the participant and the signature of an independent literate witness documented consent. Eligible contacts and contacts of contacts were informed of the outcome of the randomisation at the end of the informed consent process.

The trial personnel were predominantly composed of nationals from Guinea and other African countries. An internal quality assurance and quality control system was put in place, with 100% monitoring of study documents. An independent data and safety monitoring board (DSMB) reviewed the study protocol and the analysis plan before the analysis and assessed adverse events and efficacy results. The pilot phase of the trial began on March 23, 2015, and random assignment of clusters started on April 1, 2015. On July 31, 2015, random assignment into immediate and delayed vaccination was discontinued on the recommendation of the DSMB, whose decision took into consideration the interim analysis showing 100% vaccine efficacy^18^ (although they noted that the prespecified α spending criterion of 0·0027 was not achieved) and the low probability of being able to recruit substantial numbers of additional rings (given the declining number of cases of Ebola virus disease in the country). Thereafter, all identified rings received immediate vaccination. Ring enrolment was concluded on Jan 20, 2016.

Additionally, in view of emerging data for vaccine safety among children aged 6–17 years,^22^ the protocol was amended on Aug 15, 2015, to also include children in this age group. Consequently, we obtained written informed consent from the parents or guardians of children aged 6–17 years with written assent from children aged 12–17 years.

### Randomisation and masking

Contacts and contacts of contacts of individuals with Ebola virus disease were enumerated into clusters (and the information stored on a list) and these clusters were cluster-randomised (1:1) to either immediate vaccination or delayed vaccination (21 days later) of all eligible individuals.^20^ The teams who defined the clusters were different from the team who took informed consent or did the vaccinations. Randomisation took place only after the list enumerating all the contacts and contacts of contacts of a cluster was closed. An independent statistician not otherwise involved in the trial generated the allocation sequence, and Ebola response teams and laboratory workers were unaware of the allocation of clusters.

We used block randomisation randomly varying block sizes, stratified by location (urban *vs* rural) and size of rings (≤20 *vs* >20 individuals). The randomisation list was stored in a data management system not accessible to anyone involved in the recruitment of trial participants. Allocation of a cluster was done once the enumeration of the cluster (ie, the list of contacts and contacts of contacts) was done. Allocation of the cluster was informed to the participants at the end of the informed consent process. In the pilot phase and after July 27, 2015, clusters were not randomised and all eligible participants received the vaccine immediately after informed consent.

### Procedures

Active surveillance for, and laboratory confirmation of, cases of Ebola virus disease were independently undertaken by the national surveillance system, and cases of Ebola virus disease were confirmed by designated surveillance laboratories.^23^, ^24^ The national Ebola surveillance team and the trial team were independent; the trial team did not communicate any specific information to the surveillance teams and laboratories about which cases of Ebola virus disease were used to form a new cluster or which people would be included in a cluster.

Within 1–2 days of confirmation of a new case of Ebola virus disease, our social communication teams visited the area of residence of the case and sought the communities' consent for the trial team to enumerate a new cluster. A second team enumerated the cluster list of contacts and contacts of contacts. This list was then stored. From the complete cluster list, preliminary inclusion and exclusion criteria were applied (eg, age) to generate a list of all potential trial participants (eligible contacts and contacts of contacts) to be approached for consent. Eligible contacts and contacts of contacts cluster-randomised to immediate vaccination had only one opportunity to give their informed consent; ie, during the first contact (day 0). Eligible contacts and contacts of contacts assigned to delayed clusters had two opportunities to consent: day 0 and day 21 when vaccination was offered to the cluster.

The rVSV-ZEBOV vaccine (Merck Sharp & Dohme, Kenilworth, NJ, USA) was selected for the trial according to a framework developed by an independent group of experts.^25^ All vaccinees received one dose of 2 × 10^7^ plaque-forming units of the rVSV-ZEBOV vaccine intramuscularly in the deltoid muscle.

To assess safety, vaccinees were observed for 30 min post-vaccination and at home visits on days 3, 14, 21, 42, 63, and 84. The possible causal relationship of any adverse event to vaccination was judged by the study physicians and reported to the DSMB. Vaccinees were provided with acetaminophen or ibuprofen for the management or prevention of post-vaccination fever.

### Outcomes

The primary outcome was a laboratory confirmed case of Ebola virus disease, defined as any probable or suspected case from whom a blood sample was taken and laboratory confirmed as positive for Ebola virus; or any deceased individual with probable Ebola virus disease, from whom a post-mortem sample taken within 48 h after death was laboratory confirmed as positive for Ebola virus disease.^23^, ^24^ In our secondary objectives, we analysed the vaccine effect on deaths due to Ebola virus disease. A prespecified secondary analysis examined the overall ring vaccination effectiveness in protecting all contacts and contacts of contacts in the randomised clusters (including unvaccinated cluster members) although the trial was not powered to measure population level effects.

Local laboratories of the Ebola surveillance system confirmed cases by either detection of virus RNA by reverse transcriptase-PCR or detection of IgM antibodies directed against Ebola virus.^23^, ^24^ If available to us, aliquots of samples were retested at the European Mobile Laboratory using the RealStar Zaire Ebolavirus reverse transcriptase-PCR kit 1.0. All index cases and secondary cases of Ebola virus disease occurring in the clusters were documented using laboratory results, case investigation forms and information on chains of transmission developed independently by the national surveillance team and, if needed, supplemented with information collected by trial personnel.

A priori, we defined that only cases of Ebola virus disease with an onset 10 or more days from randomisation were valid outcomes for the trial.^18^, ^20^ This was done to account for the incubation period of Ebola virus disease,^26^, ^27^ the time between onset of symptoms and laboratory confirmation and the unknown period between vaccination and a vaccine-induced protective immune response (lag period).^20^ Additionally, vaccinated cases of Ebola virus disease with an onset of more than 31 days after random assignment were censored to account for vaccination in the delayed clusters on day 21.^18^, ^20^

### Statistical analysis

The sample size calculation is described elsewhere.^18^, ^20^ We analysed outcomes at the cluster level rather than individual level using the cumulative incidence of valid outcomes for each cluster. Additional to the planned analyses,^20^ and to address external suggestions on our interim analysis report^28^, ^29^, ^30^ we did further analyses of the randomised data. For the randomised evidence, we compared the incidence of Ebola virus disease in: 1) all vaccinated in immediate versus all contacts and contacts of contacts eligible and who consented on day 0 visit in delayed; 2) all vaccinated in immediate versus all contacts and contacts of contacts eligible in delayed; 3) all contacts and contacts of contacts eligible in immediate versus all contacts and contacts of contacts eligible in delayed; and 4) all contacts and contacts of contacts in immediate versus all contacts and contacts of contacts in delayed.

We also analysed the evidence from all clusters, including data from randomised and non-randomised clusters. For all clusters, we compared the incidence of Ebola virus disease in: all vaccinated in immediate versus all contacts and contacts of contacts who were eligible in delayed plus all contacts and contacts of contacts who were eligible but never vaccinated in immediate; all contacts and contacts of contacts in immediate versus all contacts and contacts of contacts in delayed and; all vaccinated in immediate versus all eligible but never vaccinated in immediate. Additionally, we characterised the risk of Ebola exposure and participant characteristics for all the groups being compared.

Similar to the interim analysis, if no cases of Ebola virus disease occurred in one group, we derived a 95% CI for the vaccine effect by fitting a β-binomial distribution to the cluster-level numerators and denominators and used an inverted likelihood ratio test to identify the lower bound for vaccine effect. For comparisons in which cases of Ebola virus disease occurred in both groups, we fitted a Cox proportional hazards model using a cluster-level frailty term to adjust for clustering within rings.^18^ We used Fisher's exact test to compare the proportions of clusters with at least one event across the two trial groups. The primary analysis was per protocol. We did all analyses in R, version 3.3.1.^31^ We received comments on the protocol and statistical analysis plan from an independent scientific advisory group. Independent clinical monitors validated 100% of the case report forms and an independent auditor assessed the study site, field activities, and supporting documentation. This trial is registered with the Pan African Clinical Trials Registry, number PACTR201503001057193.

### Role of the funding source

Funders other than the institutions of the authors had no role in the design of the study, data collection, data analysis, data interpretation, or writing of the report. The authors contributed to study design and data interpretation. The corresponding author had full access to all the data in the study and had final responsibility for the decision to submit for publication.

## Results

**Figure 1:**
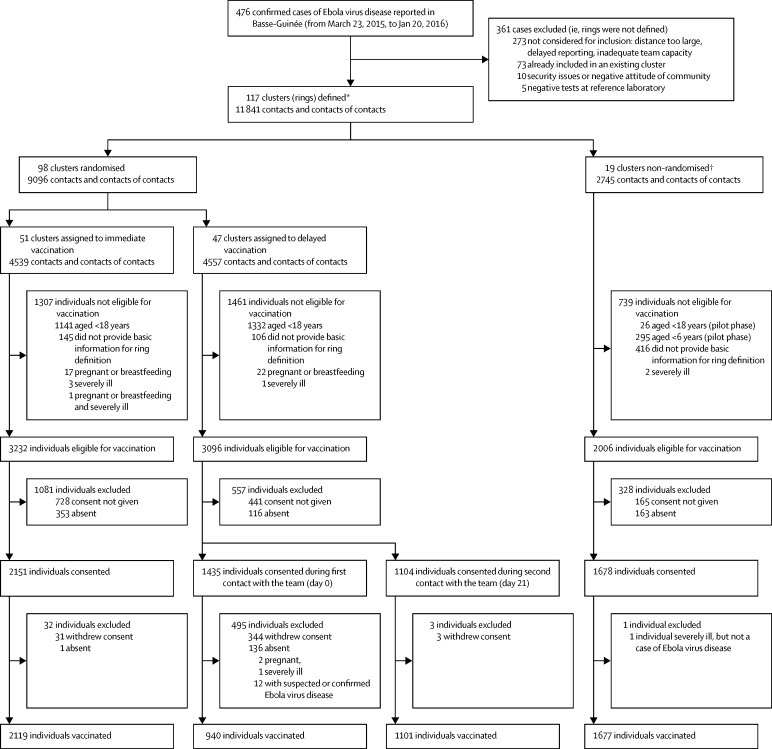
Trial profile The vaccine effects analyses set included all eligible contacts and contacts of contacts and the safety analysis set included all participants who had received the vaccine. Participants were analysed in the group corresponding to the allocated arm. *Including two non-randomised rings from Sierra Leone with 325 contacts and 255 contacts of contacts. †Including three pilot rings.

During the trial period between March 23, 2015, and Jan 20, 2016, there were 476 cases of Ebola virus disease in Guinea, all in the study area. 117 were index cases for clusters, 27 were index cases and also endpoints. In total, 105 were endpoints (75 among the eligible contacts and contacts of contacts and 30 among non-eligible contacts and contacts of contacts). We did not define a cluster around 281 (59%) of the cases of Ebola virus disease occurring during this period. These 281 cases of Ebola virus disease mostly arose during March and April, 2015, during the pilot phase and when most study teams were still being trained and the study did not have full capacity (figure 1; appendix).

In all, we obtained aliquots from 79% (93/117) Ebola virus disease index cases; 88% (30/34) of confirmed Ebola virus disease outcome cases with onset 10 or more days after randomisation and 80% (57/71) of all confirmed Ebola virus disease outcome cases. 5837 individuals in total received the vaccine (5643 adults and 194 children); all were followed up for 84 days.

The measured characteristics of index cases of Ebola virus disease and clusters were broadly comparable at baseline for immediate, delayed, and non-randomised clusters, including time from onset to randomisation and the proportion of index cases who were dead at the time of randomisation (table 1). Mean time from symptom onset in index cases to ring inclusion was 9·8 days in immediate rings, 10·9 days in delayed rings, and 7·3 days in non-randomised rings. Randomised clusters had a median 80 people (IQR 64–101) for immediate and a median 81 people (69–118) for delayed clusters. Non-randomised clusters were slightly larger with a median 105 people (49–185), partly due to public knowledge of the interim results as well as to the eligibility extension to children aged 6 years and older.

At baseline, the characteristics of contacts and contacts of contacts in all comparator groups for immediate, delayed and non-randomised clusters were largely comparable (table 2; appendix). A higher fraction of high-risk contacts was included in the immediate clusters. More than 80% of contacts and contacts of contacts were defined as contacts of contacts. Compliance with follow-up visits on all types of clusters and for all scheduled visits was more than 80% with no differences between groups (appendix).

In the randomised part of the trial, there were 4539 contacts and contacts of contacts in 51 clusters in the immediate vaccination arm (of whom 3232 were eligible, 2151 consented, and 2119 were immediately vaccinated) and 4557 contacts and contacts of contacts in 47 clusters in the delayed vaccination arm (of whom 3096 were eligible, 2539 consented and 2041 were vaccinated 21 days after randomisation; figure 1). In immediate clusters, 34% (1113/3232) of eligible individuals were not vaccinated mainly because informed consent was not obtained (n=728) or it was withdrawn (n=32), or because individuals were absent at the time of the team's visit (n=353; figure 1, Table 1, Table 2; appendix). In delayed clusters, 34% (1055/3096) of eligible individuals were not vaccinated mainly because informed consent was not obtained or it was withdrawn (n=788) or because individuals were absent at the time of the team's visit (n=252) or developed Ebola virus disease during the 0–20 days period (n=12; figure 1, Table 1, Table 2; appendix). Additionally, two individuals were pregnant, and one was severely ill, so these were not vaccinated. Among those who consented in the delayed clusters, 57% (1435/2539) gave their consent during the first visit with the study team (day 0) and 43% (1104/2539) gave consent on the vaccination visit (day 21); all were included in the cluster enumeration list.

Random assignment had little effect on the onset of Ebola virus disease during days 0–9. 20 cases of Ebola virus disease occurred among 3232 eligible contacts and contacts of contacts (nine clusters affected) in 51 immediate clusters versus 21 cases among 3096 eligible contacts and contacts of contacts (14 clusters affected) in 47 delayed clusters (table 3; appendix). However, vaccine allocation reduced Ebola virus disease onset to 0 cases from 10 days post-randomisation in immediately vaccinated contacts and contacts of contacts versus ten cases of Ebola virus disease (four clusters affected) among the eligible contacts and contacts of contacts in delayed clusters who gave consent on day 0. Vaccine efficacy was still 100% (table 3). The calculated intraclass coefficient (ICC) was high at 0·14, largely due to clustering of six confirmed endpoint cases of Ebola virus disease in one of the clusters. This would make the Fisher's test even more conservative. This ICC value contrasts with the ICC value of 0·05^20^ that we used to estimate the trial sample size and power calculation (appendix).

One additional case of Ebola virus disease was identified in the delayed clusters among eligible contacts and contacts of contacts who consented on day 21 for a total of 11 cases of Ebola virus disease among eligible and consenting contacts and contacts of contacts in delayed clusters. The remaining ten cases in the delayed clusters were among the eligible contacts and contacts of contacts who consented on day 0. Among these 11 cases of Ebola virus disease, including four vaccinees (onset 0, 2, 6, and 6 days after vaccination), seven (64%) were among unvaccinated contacts (one high-risk contact) and the four others were contacts of contacts (appendix).

The overall ring vaccination effectiveness in protecting all contacts and contacts of contacts in the randomised clusters (including unvaccinated cluster members) was 64·6% (table 3), with 65·6% of the eligible contacts and contacts of contacts receiving the vaccine at the cluster level.

No cases of Ebola virus disease occurred 10 days or more after randomisation among randomly assigned contacts and contacts of contacts vaccinated in immediate clusters versus 16 cases (7 clusters affected) among all eligible individuals in delayed clusters (table 3). Vaccine efficacy was 100% (95% CI 68·9–100·0, p=0·0045), and the calculated ICC was 0·035. Additionally, we enumerated 2745 contacts and contacts of contacts (three in the pilot phase) in 19 non-randomised clusters, 2006 of whom were eligible and 1677 were immediately vaccinated, including 194 children aged 6–17 years (figure 1).

The evidence from all 117 clusters (randomised and non-randomised) showed that no cases of Ebola virus disease occurred 10 days or more after randomisation among the 3775 immediately vaccinated contacts and contacts of contacts versus 23 cases (11 clusters affected) among the 4507 eligible contacts and contacts of contacts in delayed plus all eligible contacts and contacts of contacts never vaccinated in immediate clusters (Table 3, Table 4; appendix). Of these 23 cases of Ebola virus disease, four were vaccinated but had onset of Ebola virus disease at days 0, 2, 6, and 6 after vaccination and the remaining 19 cases were among non-vaccinated contacts and contacts of contacts. Thus, immediate vaccination resulted in complete protection against subsequent onset of Ebola virus disease 10 days later or more. The estimated vaccine efficacy here was 100% (95% CI 79·3–100·0, p=0·0033; table 4). 52% of contacts and contacts of contacts assigned to immediate vaccination and in non-randomised clusters received the vaccine immediately; vaccination protected both vaccinated and unvaccinated people in those clusters.

**Figure 2:**
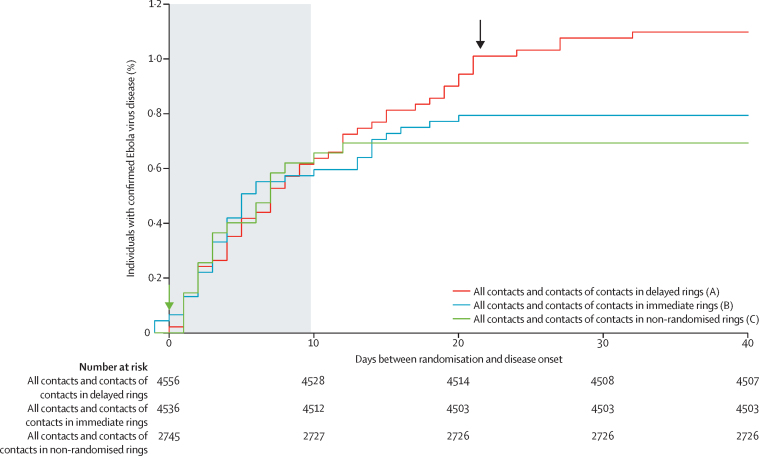
Kaplan-Meier plots for all confirmed cases of Ebola virus disease among all contacts and contacts of contacts in immediate, delayed, and non-randomised clusters Arrows show time of vaccination (at day 0 or day 21). The shaded area denotes the a priori defined lag time of 0–9 days. *Individuals aged 6–18 years were eligible for immediate vaccination in non-pilot, non-randomised rings. Description of Ebola virus disease cases 10 days or more after randomisation: A (allocated to delayed vaccination): 22 cases; six were children (aged <18 years); one was eligible and did not consent; four were absent; 11 were eligible and consented, including seven eligible and consented with illness onset on days 10–20 after randomisation plus four eligible, consented, and delayed vaccinated with onset on days 21–30 after randomisation (0, 2, 6, and 6 days after their delayed vaccination). B: ten cases, all unvaccinated; two were children (aged <18 years); four were eligible and did not consent; three were absent; one was not eligible (ie, pregnant, breastfeeding, or severely ill). C: two cases, both were children (aged <6 years and hence unvaccinated).

Cases occurred in the first 10 days after randomisation for all comparison groups, at similar times; there were no cases of Ebola virus disease among vaccinees from 10 days after randomisation or vaccination in any of the groups, with all cases arising in clusters more than 10 days post-vaccination occurring in unvaccinated individuals (figure 2). Additionally, the rVSV-ZEBOV vaccine seemed to have contributed to interrupt Ebola transmission in the clusters because no cases of Ebola virus disease among vaccinees or unvaccinated individuals were observed in immediate vaccinated clusters after 21 days after vaccination (figure 2). Details about the distribution of cases of Ebola virus disease among the various groups are in table 4 and the appendix.

Because no cases of Ebola virus disease occurred at 10 days or later in the vaccinated group, the vaccine effect was high for all the comparisons of vaccine effect on deaths due to Ebola virus disease (appendix), with 100% effect (95% CI 62·6–100, p=0·0102) when comparing all vaccinated in immediate clusters versus all eligible in delayed clusters. We were not able to do the planned secondary analyses on vaccine effect against probable and suspected cases because of near-universality of laboratory testing of such cases in Guinea during the study period, leaving only 26/502 (5%) of cases without a definitive diagnosis. Five cases of Ebola virus disease initially considered as index cases for clusters were negative by confirmatory retesting and the corresponding clusters were therefore excluded from the analysis. No endpoint cases tested negative on confirmatory retesting.

**Figure 3:**
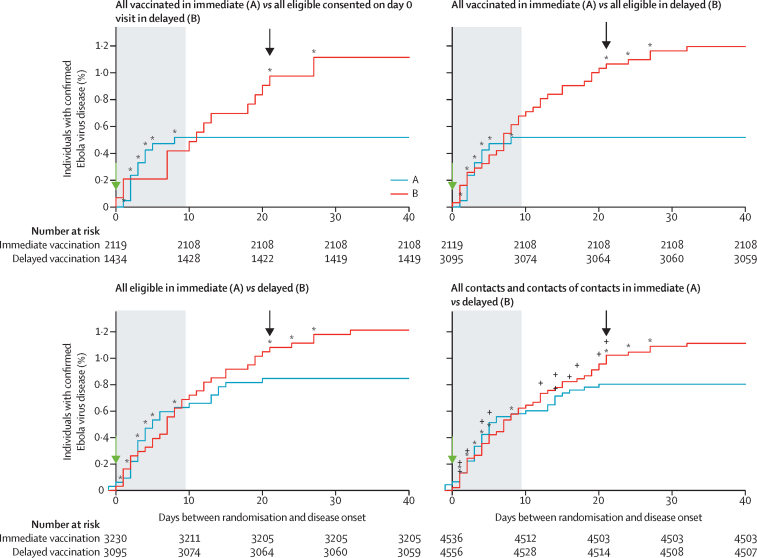
Kaplan-Meier plots for confirmed cases of Ebola virus disease in different study populations Arrows show time of vaccination (at day 0 or day 21); the plus signs denote cases among non-eligible children and the stars denote cases among vaccinated individuals; the shaded area denotes the a priori defined lag time of 0–9 days.

In total, we identified 105 cases of Ebola virus disease among all contacts and contacts of contacts (eligible or not for vaccination) in the 117 clusters defined (98 randomised clusters and 19 non-randomised clusters). The overall attack rate was 0·9% (95% CI 0·7–1·1) considering the 105 cases occurring among 11 841 individuals enumerated in 117 rings. None of the cases occurred in vaccinated individuals 10 days or more after being vaccinated (figure 3; appendix).

Moreover, when comparing all contacts and contacts of contacts in clusters immediately vaccinated versus all contacts and contacts of contacts in delayed clusters plus all contacts and contacts of contacts never vaccinated in immediate or non-randomised clusters, vaccine protection was 100% (table 3) further indicating that the vaccine is highly protective (table 4; appendix). This represents the totality of evidence for high vaccine efficacy when comparing all immediately vaccinated people to all delayed or unvaccinated people. The overall ring vaccination effectiveness in protecting all contacts and contacts of contacts (including vaccinated and unvaccinated cluster members) was 70·1% (table 3) with 52·1% (3796/7284) of the contacts and contacts of contacts vaccinated.

Cases occurred in the first 10 days at a similar time in immediate, delayed, and non-randomised clusters and all comparison groups. There were no cases of Ebola virus disease among vaccinees from 10 days post-vaccination in any of the groups (figure 3, appendix). Moreover, rVSV-ZEBOV vaccine contributed to interrupt Ebola transmission with no cases of Ebola virus disease after 32 days after randomisation in randomly assigned and non-randomly assigned clusters in vaccinated and non-vaccinated individuals (Figure 2, Figure 3).

3149 (53·9%) of 5837 individuals reported at least one adverse event in the 14 days after vaccination (appendix); across all adverse events, solicited and unsolicited, 87·5% (6311/7211) were mild, 11·0% (793/7211) moderate, and 1·2% (83/7211) severe (appendix). Across all age groups, headache (1832 [25·4%]), fatigue (1361 [18·9%]), and muscle pain (942 [13·1%) were the most commonly reported adverse events in this period across all age groups. Data from children indicated that in the 3 days after vaccination, by percentage of individuals with the events, the commonly reported adverse events were headache (51/97 [52·6%]), fatigue (11/97 [11·3%]), and injection pain (9/97 [9·3%]). Adults most commonly reported headache (1781/7114 [25·0%]), fatigue (1350/7114 [19·0%]), and muscle pain (937/7114 [13·2%]) in the same period. Arthralgia was the fourth most reported adverse event (table 5; reported by 17·9% of vaccinated participants), and was reported in 4/180 (2·2%) of vaccinated children with a mean duration of 4·5 days (IQR 3–5) and in 915/4960 (18·5%) of vaccinated adults with a mean duration of 2 days (2–4). Cases resolved spontaneously without sequelae.

80 serious adverse events were reported. The most common diagnosis was Ebola virus disease in 39/80 participants (48·7%) followed by road traffic accident injury in 4/80 (5%; appendix). Two serious adverse events were judged to be related to vaccination (a febrile reaction and one anaphylaxis, which resolved without sequelae) and one possibly related (influenza-like illness) which also recovered without sequelae. 15 serious adverse events occurred among enrolled but non-vaccinated participants; 14 were Ebola virus disease in participants (all with onset 0–10 days after randomisation) and one was a road traffic accident injury.

## Discussion

The results presented in this final analysis of our Ebola ça Suffit trial strengthen the interim estimates and conclusions^18^ that the rVSV-ZEBOV vaccine has high protective efficacy and effectiveness to prevent Ebola virus disease. The current report included data from 27 additional clusters; eight of which were randomly assigned to immediate or delayed vaccination. No vaccinees developed Ebola virus disease 10 days or more after randomisation, but cases occurred in unvaccinated comparators, both in randomised and non-randomised clusters. When we compared randomly assigned contacts and contacts of contacts vaccinated in immediate clusters (day 0) versus all eligible in delayed clusters, vaccine efficacy was 100%. These final analyses hence support the interim report efficacy results, indicating that ring vaccination with an effective vaccine can contribute as a control strategy for future outbreaks of Ebola virus disease.

Data from early phase 1–2 studies suggest that rVSV-ZEBOV is well tolerated in human beings and produces a rapid immune response after a single dose,^32^, ^33^ with its short-term protection most likely mediated by innate immunity. One explanation for this finding is that innate immune activation by the vaccine might provide a window of protection that restricts virus replication in the essential period needed for the development of specific adaptive responses.^11^

A devastating outbreak of Ebola virus disease is clearly not the ideal situation for doing a vaccine trial. The health-care system in Guinea was strained, potential trial participants were worried about a candidate vaccine made by foreign people, and the Ebola virus disease response teams were facing security issues. Therefore, we made a deliberate decision to tailor the logistical implementation of the trial to local conditions.^20^ The close collaboration with, and the support from, the Guinean National Authorities was a catalysing factor in the successful implementation of the trial. In addition, we made efforts to ensure full ownership and understanding by national authorities and communities through active community engagement and individual consent. Despite the challenges, our team was able to do the trial in compliance with good clinical practice and international standards.

We addressed common biases of cluster-randomised trials. Our analyses suggested no imbalances in the demographic characteristics of the index cases or the risk factors for Ebola virus disease infection documented in the contacts and contacts of contacts, further supporting the hypothesis that any differences were due to a vaccine effect. A few differences remained between groups. Time to cluster definition was slightly shorter in the immediate vaccination group, which also had more high-risk contacts reported. All valid clusters enrolled were analysed, and more than 90% of vaccinees were followed up in all groups. To address recruitment bias, we finalised and closed the enumeration of eligible contacts and contacts of contacts in each cluster before cluster allocation. Although we implemented prospective recruitment, only contacts and contacts of contacts included in the cluster enumeration list were given the opportunity to provide informed consent. A different team obtained informed consent to minimise subversion. Participants were informed of the outcome of randomisation at the end of the informed consent process, and both immediate and delayed clusters were given identical information about the trial before consent.

The inclusion of temporarily absent contacts and contacts of contacts contributed to a moderate within-cluster percentage of vaccinees among the eligible contacts and contacts of contacts of 65·6% in immediately randomised clusters, 65·9% in delayed randomised clusters and 83·6% in non-randomised clusters. The higher uptake of vaccine among the contacts and contacts of contacts in non-randomised clusters might be attributable to public knowledge of the interim results as well as the inclusion of children aged 6 years and older.

Confirmation of cases with Ebola virus disease was done independently of the study team as part of the national surveillance of Ebola virus disease, throughout and beyond the follow-up period of the trial. Confirmatory retesting of samples of index cases and endpoints augmented the independence of the process.

Although eligible individuals in the delayed arm had two opportunities to consent (day 0 and day 21), those consenting at day 21 could only do so if they had not been diagnosed with Ebola virus disease in the intervening time. We therefore also presented a comparison of the vaccine effect with individuals in the delayed group who gave consent during the first visit (day 0). Because only one additional case of Ebola virus disease was documented among those consenting late (on day 21), the estimated vaccine effect remained 100% but the lower 95% CI bound changed from 68·9% to 63·5%.

These results are the only efficacy data available for rVSV-ZEBOV, and for any Ebola virus disease vaccine, available from trials in human beings to date. Because of the challenges of implementing the trial, we decided not to attempt to collect biological samples from vaccinees for immunological analysis and therefore an individual-level correlation of protection analysis was unfortunately not possible. Such interpretation would also have been rendered difficult given that there were no break-through cases among vaccinees after day 10. The high levels of vaccine effect noted in this study are in line with findings from other studies, such as the phase 2 PREVAIL trial,^34^ which used the same dose and route of administration and showed that 94% of 500 individuals who received the rVSV-ZEBOV vaccine seroconverted after a month. Results from animal studies with rVSV-ZEBOV vaccines have also shown consistently high and rapid protection.^11^, ^12^ Our results will be further complemented by those from a cohort study to assess immune response after vaccination that we did in front-line workers in Guinea.

We designed this trial to have a high probability of generating meaningful data for the efficacy of the vaccine despite the low and declining incidence of Ebola virus disease. Our design attempted to address the challenge that the comparator group should not be denied access (at least indefinitely) to the experimental vaccine, an issue raised by ethics committees and others, and we opened eligibility for children as soon as preliminary safety data were available from phase 1 studies.^22^ In our final phase 3 analyses no serious safety signals were identified in children or adults.

A feature of the ring vaccination trial design is the potential to measure indirect protection within the clusters. Our data suggest that such indirect effect occurred, but the small sample size prevented a definitive conclusion. Nevertheless, the high efficacy of the rVSV-ZEBOV vaccine, as indicated by the randomised and non-randomised analysis, suggests that the Ebola ça Suffit trial itself had some contribution to foreshortening the epidemic of Ebola virus disease in west Africa by direct and indirect aversion of cases. The evidence from randomised and non-randomised clusters and the fact no cases of Ebola virus disease occurred 10 or more days after vaccination (through the 84 days follow-up period and from the indefinite surveillance system throughout the epidemic period) indicates substantial protection of rVSV-ZEBOV against Ebola virus disease. Ring vaccination was effective in contributing to controlling the Ebola virus disease outbreak. Results from mathematical modelling studies, which used the data from the ring vaccination trial, indicate that using ring vaccination within a surveillance and containment strategy could be highly effective in controlling future outbreaks of Ebola virus disease.^35^ The findings from Ebola ça Suffit showed that it is feasible to undertake efficacy trials in the challenging circumstances of epidemics. Vaccine trial designs using case-reactive strategies similar to those of the ring vaccination trial might have an application in future haemorrhagic fever outbreaks and in other infectious disease epidemics.

**This online publication has been corrected. The first corrected version appeared at thelancet.com on December 23, 2016. The second corrected version appeared on February 2, 2017**

## Figures and Tables

**Table 1:** Baseline characteristics of clusters and index cases

	**Randomised**		**Not randomised**	
	Assigned to immediate vaccination (51 clusters)	Assigned to delayed vaccination (47 clusters)	Assigned to immediate vaccination (19 clusters)	All clusters (117 clusters)
**Index cases used to define clusters**				
Age (years)	35 (18–43)	35 (27–50)	23 (13–42)	35 (20–47)
Women	27/51 (53%)	31/47 (66%)	12/19 (63%)	70/117 (60%)
Dead at time of randomisation	30/51 (59%)	32/47 (68%)	9/19 (47%)	71/117 (61%)
Time from onset of symptoms to admission to hospitalisation or isolation (days)	3·9 (2·9)	3·8 (2·6)	3·2 (2·4)	3·7 (2·7)
Time from onset of symptoms for index cases to randomisation of cluster (days)	9·7 (5·3)	11 (4·1)	..	10·3 (4·8)
Time from onset of symptoms for index cases to inclusion of cluster (days)	9·8 (5·1)	10·9 (4·1)	7·3 (3·7)	9·9 (4·6)
**Characteristics of clusters**				
Located in rural areas	39/51 (76%)	36/47 (77%)	9/19 (47%)	84/117 (72%)
Total number of people in cluster	80 (64–101)	81 (69–118)	105 (49–185)	83 (66–115)

Data are median (IQR), n/N (%), or mean (SD). ..=not applicable.

**Table 2:** Baseline characteristics of eligible contacts and contacts of contacts

	**Randomly assigned**					Not randomly assigned*		**Totality of evidence**	
	Assigned to immediate vaccination (51 clusters, n=3232)		Assigned to delayed vaccination (47 clusters, n=3096)			Assigned to immediate vaccination (19 clusters, n=2006)		All clusters (117 clusters, n=8334)	
	Consent	No consent	Consent visit day 0†	Consent visit day 21†	No consent	Consent	No consent	Immediately vaccinated	Delayed or never vaccinated
**Individuals' characteristics**									
Number of individuals	2151	1081	1435	1104	557	1678	328	3796	4538
Age (years)	40 (29–55)	30 (25–45)	39 (27–53)	37 (27–50)	32 (23–45)	30 (22–44)	25 (18–35)	35 (25–50)	35 (25–50)
Women	640/2151 (30%)	608/1081 (56%)	428/1434 (30%)	404/1104 (37%)	319/557 (57.3%)	593/1678 (35%)	179/328 (54.6%)	1223/3796 (32%)	1948/4537 (43%)
**Contacts with index cases**									
No detailed contact information (no consent)	0/2151	1081/1081 (100%)	0/1435	0/1104	557/557 (100%)	0/1678	328/328 (100%)	0/3796	1966/4538 (43%)
Contact of contact‡	1727/2151 (80%)	..	1160/1435 (81%)	971/1104 (88%)	..	1418/1678 (85%)	..	3116/3796 (82%)	2160/2572 (84%)
Contact‡	424/2151 (20%)	..	275/1435 (19%)	133/1104 (12%)	..	260/1678 (15%)	..	680/3796 (18%)	412/2572 (16%)
High-risk contact‡	330/2151 (15%)	..	171/1435 (12%)	58/1104 (5%)	..	246/1678 (15%)	..	574/3796 (15%)	231/2572 (9%)

Data are median (IQR) or n/N (%). ..=data not available.

* Six non-randomised rings included children aged 6 years and older (n=273).

† Informed consent was obtained either during the first visit (day 0) or the second visit (day 21) of the trial team.

‡ Proportion calculated among individuals with available contact information. Two individuals were pregnant and one was severely ill.

**Table 3:** Effect of vaccine on cases of Ebola virus disease in different study populations

	All clusters*				**Randomised clusters**†			
	1	2	3	4	5	6	7	8
	All vaccinated in immediate (group A) *vs* all contacts and contacts of contacts in delayed plus all never-vaccinated in immediate or non-randomised (group B)	All vaccinated in immediate (group A) *vs* all eligible in delayed plus all eligible never-vaccinated in immediate (group B)	All contacts and contacts of contacts in immediate (group A) *vs* delayed (group B)	All vaccinated in immediate (group A) *vs* all eligible never vaccinated in immediate (group B)	All vaccinated in immediate (group A) *vs* all eligible and consented on day 0 visit in delayed (group B)	All vaccinated in immediate (group A) *vs* all eligible in delayed (group B)	All eligible in immediate (group A) *vs* all eligible delayed (group B)	All contacts and contacts of contacts in immediate (group A) *vs* all contacts and contacts of contacts in delayed (group B)
**Group A**								
Number of individuals (clusters)	3775 (70)	3775 (70)	7241 (70)	3775 (70)	2108 (51)	2108 (51)	3212 (51)	4513 (51)
Cases of Ebola virus disease (clusters affected)	0 (0)	0 (0)	12 (7)	0 (0)	0 (0)	0 (0)	7 (4)	10 (5)
Attack rate	0%	0%	0·17%	0%	0%	0%	0·22%	0·22%
**Group B**								
Number of individuals (clusters)	7995 (116)	4507 (104)	4529 (47)	1432 (57)	1429 (46)	3075 (47)	3075 (47)	4529 (47)
Cases of Ebola virus disease (clusters affected)	34 (15)	23 (11)	22 (8)	7 (4)	10 (4)	16 (7)	16 (7)	22 (8)
Attack rate	0·43%	0·51%	0·49%	0·49%	0·7%	0·52%	0·52%	0·49%
Vaccine effect								
Vaccine efficacy/effectiveness‡ (%, 95% CI)	100% (77·0 to 100·0)	100% (79·3 to 100·0)	70·1% (−4·9 to 91·5)	100% (−51·5 to 100·0)	100% (63·5 to 100·0)	100% (68·9 to 100·0)	64·6% (−46·5 to 91·4)	64·6% (−44·2 to 91·3)
p value§	0·0012	0·0033	0·2759	0·125	0·0471	0·0045	0·344	0·3761

* Randomly assigned and non-randomly assigned individuals who were allocated to immediate vaccination were combined.

† Non-randomised immediate clusters are excluded from this analysis.

‡ From fitting a β-binomial distribution to the cluster-level numerators and denominators and using an inverted likelihood ratio test to identify the lower bound for vaccine efficacy (columns 1, 2, 5, and 6); from a Cox proportional hazards model (column 3, 7, and 8); from signed test (two-sided): probability of observing endpoints in control groups among treatment–control mismatched pairs and under the null hypothesis that the vaccine has no efficacy (column 4).

§ From Fisher's exact test (two-sided), which is approximate for columns 1 and 2. From signed test (two-sided): probability of observing endpoints in control groups among treatment–control mismatched pairs and under the null hypothesis that the vaccine has no efficacy (column 4).

**Table 4:** Distribution of confirmed cases of Ebola virus disease among enumerated contacts and contacts of contacts in all clusters

		**Eligible adults assigned to immediate vaccination**		**All eligible adults assigned to delayed vaccination**	**Eligible adults not assigned**		Non-eligible*participants (not vaccinated)		
		Immediately Vaccinated	Never vaccinated		Immediately Vaccinated	Never vaccinated	All assigned to immediate vaccination	All assigned to delayed vaccination	All not assigned
Contacts and contacts of contacts (clusters)		2119 (51)	1113 (48)	3096 (47)	1677 (19)	329 (10)	1307 (50)	1461 (47)	739 (19)
Attack rates									
	Overall	11/2119 (0·5%)	16/1113 (1·4%)	37/3096 (1·2%)	10/1677 (0·6%)	1/329 (0·3%)	9/1307 (0·7%)	13/1461 (0·9%)	8/739 (1·1%)
	Onset <10 days since being randomly assigned	11/2111 (0·5%)	9/1113 (0·8%)	21/3096 (0·7%)	10/1677 (0·6%)	1/329 (0·3%)	6/1307 (0·5%)	7/1461 (0·5%)	6/739 (0·8%)
	Onset ≥10 days since being randomly assigned	0/2108	7/1104 (0·6%)	16/3075 (0·5%)	0/1667	0/328	3/1301 (0·2%)	6/1454 (0·4%)	2/733 (0·3%)
Clusters affected by cases with onset ≥10 days after being randomly assigned									
	0 cases	51/51 (100%)	44/48 (91·7%)	40/47 (85·1%)	19/19 (100%)	10/10 (100%)	48/50 (96%)	44/47 (93·6%)	17/19 (89·5%)
	1 case	..	2/48 (4·2%)	3/47 (6·4%)	..	..	1/50 (2%)	2/47 (4·3%)	2/19 (10·5%)
	2 cases	..	1/48 (2·1%)	2/47 (4·3%)	..	..	1/50 (2%)	..	..
	3 cases	..	1/48 (2·1%)	1/47 (2·1%)	..	..	..	..	..
	4 cases	..	..	..	..	..	..	1/47 (2·1%)	..
	6 cases	..	..	1/47 (2·1%)	..	..	..	..	..

* Aged <18 years, pregnant, or lactating (full list of exclusion criteria in 19, 20). ..=data not available.

**Table 5:** Frequency of solicited adverse events by time since vaccination in children and adults.

	**0–30 min**	**31 min to 3 days**	**4–14 days**
**Children aged between 6–<18 years (n=194)**			
Arthralgia	0	3 (3·5%)	1 (9.1%)
Diarrhoea	0	0	1 (9·1%)
Fatigue	0	10 (11·6%)	1 (9·1%)
Fever	0	1 (1·2%)	1 (9·1%)
Headache	0	47 (54·7%)	4 (36·4%)
Induration	0	0	0
Injection pain	0	9 (10·5%)	0
Muscle pain	0	4 (4·7%)	1 (9·1%)
Myalgia	0	4 (4·7%)	1 (9·1%)
Vomiting	0	1 (1·2%)	0
Other adverse events	0	7 (8·1%)	1 (9·1%)
Total	0	86 (100·0%)	11 (100·0%)
**Adults aged 18 years and older (n=5643)**			
Arthralgia	3 (2%)	851 (13·5%)	79 (12·3%)
Diarrhoea	0	53 (0·8%)	15 (2·3%)
Fatigue	5 (3·3%)	1233 (19·5%)	112 (17·4%)
Fever	2 (1·3%)	8 (0·1%)	2 (0·3%)
Headache	41 (27·3%)	1563 (24·7%)	177 (27·5%)
Induration	0	1 (<1%)	0
Injection pain	70 (46·7%)	362 (5·7%)	8 (1·2%)
Muscle pain	7 (4·7%)	875 (13·8%)	55 (8·5%)
Myalgia	6 (4·0%)	816 (12·9%)	47 (7·3%)
Vomiting	0	21 (0·3%)	4 (0·6%)
Other adverse events	16 (10·7%)	537 (8·5%)	145 (22·5%)
Total	150 (100·0%)	6320 (100·0%)	644 (100·0%)

Data are n (%); individuals might have had more than one adverse event.
