# Department of Error

**DOI:** 10.1016/S0140-6736(17)30210-6

**Published:** 2017-02-04

*Henao-Restrepo AM, Camacho A, Longini IM, et al. Efficacy and effectiveness of an rVSV-vectored vaccine in preventing Ebola virus disease: final results from the Guinea ring vaccination, open-label, cluster-randomised trial (Ebola Ça Suffit!).* Lancet *2016; **389:** 505–18*—In this Article, the UK Government through the Department for International Development should be included in the list of funders. Additionally, some of the reference number assignments have changed, and some names have been added to the Acknowledgments section. The second corrected version appeared at thelancet.com on Feb 2, 2017, and the printed Article is correct.

