# Department of Error

**DOI:** 10.1016/S0140-6736(16)32633-2

**Published:** 2017-02-04

*Henao-Restrepo AM, Camacho A, Longini IM, et al. Efficacy and effectiveness of an rVSV-vectored vaccine in preventing Ebola virus disease: final results from the Guinea ring vaccination, open-label, cluster-randomised trial (Ebola Ça Suffit!).* Lancet *2016; **389:** 505–18*—In this Article, the following corrections have been made: axes corrected in figure 2, reordered reference listing at the end of the paper, duplicated ‘that’ word removed from page 1 and page 8, the number ‘46·6%’ changed to ‘65·6%’ on page 7, the words ‘30 days’ changed to ‘32 days’ on page 9, and the word ‘vaccines’ changed to ‘vaccinees’ on page 11. The first corrected version appeared at thelancet.com on Dec 23, 2016, and the printed Article is correct.

