# Supplementary material for: Efficacy and effectiveness of an rVSV-vectored vaccine in preventing Ebola virus disease: final results from the Guinea ring vaccination, open-label, cluster-randomised trial (Ebola Ça Suffit!)
Source: Lancet. 2017 Feb 4;389(10068):505–18. doi: 10.1016/S0140-6736(16)32621-6 (PMC5364328; doi:10.1016/S0140-6736(16)32621-6)
Supplement: Supplementary appendix [file mmc1.pdf]

THE LANCET

Supplementary appendix

This appendix formed part of the original submission and has been peer reviewed. We post it as supplied by the authors. **This online publication has been corrected. The corrected version first appeared at thelancet.com on December 23, 2016**.

Supplement to: Henao-Restrepo AM, Camacho A, Longini IM, et al. Efficacy and effectiveness of an rVSV-vectored vaccine in preventing Ebola virus disease: final results from the Guinea ring vaccination, open-label, cluster-randomised trial (Ebola Ça Suffit!). *Lancet* 2016; published online Dec 22. http://dx.doi.org/10.1016/S0140-6736(16)32621-6.

Table S1: Compliance with follow-up visits for safety monitoring for different study populations.

	Randomised					Not randomised*		All clusters	
	Allocated to immediate vaccination (51 clusters, n=3232)		Allocated to delayed vaccination (47 clusters, n=3096)			Allocated to immediate vaccination (19 clusters, n=2006)		All clusters (117 clusters, n=8334)	
	Consent	No consent	Consent visit D0†	Consent visit D21†	No consent	Consent	No consent	Immediately vaccinated	Delayed or never vaccinated
**Compliance with follow-up visits for safety monitoring among eligible and consenting CCCs**									
Day 3	1958/2118 (92.4%)	-	916/1058 (86.6%)	1048/1101 (95.2%)	-	1639/1676 (97.8%)	-	3597/3793 (94.8%)	1964/2160 (90.9%)
Day 14	1819/2107 (86.3%)	-	876/1055 (83%)	1022/1100 (92.9%)	-	1564/1668 (93.8%)	-	3383/3774 (89.6%)	1898/2156 (88%)
Day 21	1911/2107 (90.7%)	-	877/1054 (83.2%)	1001/1100 (91%)	-	1567/1668 (93.9%)	-	3478/3774 (92.2%)	1878/2155 (87.1%)
Day 42	1895/2098 (90.3%)	-	873/947 (92.2%)	997/1099 (90.7%)	-	1552/1663 (93.3%)	-	3447/3761 (91.7%)	1870/2046 (91.4%)
Day 63	1884/2086 (90.3%)	-	879/939 (93.6%)	973/1093 (89%)	-	1541/1659 (92.9%)	-	3425/3745 (91.5%)	1852/2032 (91.1%)
Day 84	1934/2070 (93.4%)	-	887/933 (95.1%)	1015/1085 (93.5%)	-	1574/1659 (94.9%)	-	3508/3729 (94.1%)	1902/2018 (94.3%)

*6 non-randomised rings included children 6 years of age and older (n=273).
†Informed consent was obtained either during the first visit (day 0) or second visit (day 21) of trial team.

Table S2: Distribution of all confirmed EVD cases among all eligible CCCs* in various types of clusters according to informed consent status and onset of EVD cases (outcomes) since randomisation**

	Randomised					Not randomised†		All clusters	
	Allocated to immediate vaccination (51 clusters, n=3232)		Allocated to delayed vaccination (47 clusters, n=3096)			All immediately vaccinated (19 clusters, n=2006)		All clusters (117 clusters, n=8334)	
	Consent	No consent	Consent visit D0‡	Consent visit D21‡	No consent	Consent	No consent	Immediately vaccinated	Delayed or never vaccinated
**Eligible CCCs**									
Number of subjects (clusters)	2151[a] (51)	1081 (48)	1435 (46)	1104 (45)	557 (35)	1678[b] (19)	328 (10)	3796 (70)	4538 (105)
Mean (SD) time from randomisation§ to consent, in days	1.46 (1.23)	-	1.71 (1.65)	21.72 (1.25)	-	2.47 (3.24)	-	1.91 (2.39)	10.28 (10.03)
**Immediately vaccinated subjects**									
Number immediately vaccinated (clusters)	2119[a] (51)	-	-	-	-	1677[a] (19)	-	3796 (70)	-
Mean (SD) time from randomisation§ to immediate vaccination, in days	1.58 (1.13)	-	-	-	-	2.47 (3.24)	-	1.97 (2.35)	-
**Confirmed EVD cases <10 days since randomisation§**									
Number of EVD cases (affected clusters)	11 (4)	9 (7)	6 (5)	-	15 (10)	10 (7)	1 (1)	21 (11)	31 (22)
Contact with index case									
No detailed contact information (no consent)	0/11 (0%)	9/9 (100%)	0/6 (0%)	-	15/15 (100%)	0/10 (0%)	1/1 (100%)	0/21 (0%)	25/31 (80.6%)
Contact of contact	1/11 (9.1%)	-	0/6 (0%)	-	-	0/10 (0%)	-	1/21 (4.8%)	0/6 (0%)
Contact	10/11 (90.9%)	-	6/6 (100%)	-	-	10/10 (100%)	-	20/21 (95.2%)	6/6 (100%)
High-risk contact	10/11 (90.9%)	-	5/6 (83.3%)	-	-	10/10 (100%)	-	20/21 (95.2%)	5/6 (83.3%)
**Confirmed EVD cases ≥10 days since randomisation§**									
Number of EVD cases (affected clusters)	0 (0)	7 (4)	10[c] (4)	1[d] (1)	5 (4)	0 (0)	0 (0)	0 (0)	23 (11)
Contact with index case									
No detailed contact information (no consent)	-	7/7 (100%)	0/10 (0%)	0/1 (0%)	5/5 (100%)	-	-	-	12/23 (52.2%)
Contact of contact	-	-	3/10 (30%)	1/1 (100%)	-	-	-	-	4/11 (36.4%)
Contact	-	-	7/10 (70%)	0/1 (0%)	-	-	-	-	7/11 (63.6%)
High-risk contact	-	-	1/10 (10%)	0/1 (0%)	-	-	-	-	1/11 (9.1%)

*see full list of exclusion criteria [20]
**reporting of EVD cases was independent of participation in the trial and was done independently by the national surveillance system
†6 non-randomised rings included children 6 - 17 years of age (n=273)
‡Informed consent for enumerated and eligible subjects was obtained either during Day 0 or Day 21 contact with trial team
§For non-randomised rings the date of inclusion of the ring was used.
a 32 eligible and consent subjects, allocated to immediate clusters, were not vaccinated. None was an EVD case.
b 1 non-randomised eligible and consent subject was not vaccinated. He was not an EVD case.
c Including 3 vaccinees with onset of symptoms 0-9 days after vaccination (days 0, 6 and 6 days after vaccination)
d Vaccinee with onset of symptoms 0-9 days after vaccination (2 days after vaccination)

Table S3: Distribution of confirmed Ebola virus disease cases in vaccinated and unvaccinated CCCs in all clusters (immediate, delayed and not randomised clusters)

	Eligible CCCs					Non-eligible CCCs*		
	Allocated immediate vaccination		Allocated delayed vaccination	Not randomised		Allocated immediate vaccination	Allocated delayed vaccination	Not Randomised
	**Vaccinated immediately (n=2119)**	**Never vaccinated (n=1113)**	**All (n=3096)**	**Vaccinated immediately (n=1677)**	**Never vaccinated (n=329)**	**All (n=1307)**	**All (n=1461)**	**All (n=739)**
**Allocated immediate (47 clusters)**								
Cluster I1	4 - 0	2 - 3	-	-	-	0 - 1	-	-
Cluster I2	0 - 0	2 - 2	-	-	-	1 - 0	-	-
Cluster I3	0 - 0	1 - 1	-	-	-	1 - 0	-	-
Cluster I4	5 - 0	1 - 0	-	-	-	1 - 2	-	-
Cluster I5	0 - 0	1 - 0	-	-	-	1 - 0	-	-
Cluster I6	0 - 0	1 - 0	-	-	-	0 - 0	-	-
Cluster I7	0 - 0	1 - 0	-	-	-	0 - 0	-	-
Cluster I8	0 - 0	0 - 1	-	-	-	0 - 0	-	-
Cluster I9	1 - 0	0 - 0	-	-	-	0 - 0	-	-
Cluster I10	1 - 0	0 - 0	-	-	-	0 - 0	-	-
Cluster I11	0 - 0	0 - 0	-	-	-	2 - 0	-	-
40 clusters with 0 cases	-	-	-	-	-	-	-	-
**Allocated delay (51 clusters)**								
Cluster D1	-	-	3 - 3	-	-	-	0 - 4	-
Cluster D2	-	-	2 - 2	-	-	-	0 - 0	-
Cluster D3	-	-	2 - 0	-	-	-	2 - 0	-
Cluster D4	-	-	2 - 0	-	-	-	1 - 0	-
Cluster D5	-	-	2 - 0	-	-	-	0 - 0	-
Cluster D6	-	-	2 - 0	-	-	-	0 - 0	-
Cluster D7	-	-	1 - 6	-	-	-	2 - 1	-
Cluster D8	-	-	1 - 2	-	-	-	0 - 0	-
Cluster D9	-	-	1 - 0	-	-	-	1 - 1	-
Cluster D10	-	-	1 - 0	-	-	-	1 - 0	-
Cluster D11	-	-	1 - 0	-	-	-	0 - 0	-
Cluster D12	-	-	1 - 0	-	-	-	0 - 0	-
Cluster D13	-	-	1 - 0	-	-	-	0 - 0	-
Cluster D14	-	-	1 - 0	-	-	-	0 - 0	-
Cluster D15	-	-	0 - 1	-	-	-	0 - 0	-
Cluster D16	-	-	0 - 1	-	-	-	0 - 0	-
Cluster D17	-	-	0 - 1	-	-	-	0 - 0	-
30 clusters with 0 cases	-	-	-	-	-	-	-	-
**Not randomised (19 clusters)**								
Cluster N1	-	-	-	2 - 0	1 - 0	-	-	2 - 0
Cluster N2	-	-	-	2 - 0	0 - 0	-	-	2 - 0
Cluster N3	-	-	-	1 - 0	0 - 0	-	-	2 - 1
Cluster N4	-	-	-	1 - 0	0 - 0	-	-	0 - 0
Cluster N5	-	-	-	1 - 0	0 - 0	-	-	0 - 0
Cluster N6	-	-	-	2 - 0	-	-	-	0 - 0
Cluster N7	-	-	-	1 - 0	-	-	-	0 - 1
12 clusters with 0 cases	-	-	-	-	-	-	-	-
**Attack rates**								
Onset <10 days since randomisation	11/2119 (0.5%)	9/1113 (0.8%)	21/3096 (0.7%)	10/1677 (0.6%)	1/329 (0.3%)	6/1307 (0.5%)	7/1461 (0.5%)	6/739 (0.8%)
Onset ≥10 days since randomisation	0/2108 (0%)	7/1104 (0.6%)	16/3075 (0.5%)	0/1667 (0%)	0/328 (0%)	3/1301 (0.2%)	6/1454 (0.4%)	2/733 (0.3%)
**Total**	**11/2119 (0.5%)**	**16/1113 (1.4%)**	**37/3096 (1.2%)**	**10/1677 (0.6%)**	**1/329 (0.3%)**	**9/1307 (0.7%)**	**13/1461 (0.9%)**	**8/739 (1.1%)**

Data X-Y are X: number of confirmed cases with onset <10 days since randomization; Y: number of confirmed cases with onset ≥10 days since randomisation.
*see full list of exclusion criteria in [18].

Table S4: Vaccine effect on EVD deaths* for different comparisons of study population.

	All clusters**				Randomised clusters***			
	**All vaccinated in immediate (A) versus all delayed plus all never vaccinated in immediate (B)**	**All vaccinated in immediate (A) versus all eligible in delayed plus all eligible never vaccinated in immediate (B)**	**All contacts and contacts of contacts in immediate (A) versus delayed (B)**	**All vaccinated in immediate (A) versus all eligible never vaccinated in immediate (B)**	**All vaccinated in immediate (A) versus all eligible and consented on D0 visit in delayed (B)**	**All vaccinated in immediate (A) versus all eligible in delayed (B)**	**All eligible in immediate (A) versus delayed (B)**	**All CCCs in immediate (A) versus all CCCs in delayed (B)**
**Group A**								
Participants (clusters)	3775 (70)	3775 (70)	7241 (70)	3775 (70)	2108 (51)	2108 (51)	3212 (51)	4513 (51)
EVD cases (clusters affected)	0 (0)	0 (0)	2 (2)	0 (0)	0 (0)	0 (0)	1 (1)	2 (2)
Risk of EVD-specific death	0%	0%	0.03%	0%	0%	0%	0.03%	0.04%
**Group B**								
Participants (clusters)	7995 (116)	4507 (104)	4529 (47)	1432 (57)	1429 (46)	3075 (47)	3075 (47)	4529 (47)
EVD cases (clusters affected)	17 (8)	13 (7)	15 (6)	1 (1)	8 (4)	12 (6)	12 (6)	15 (6)
Risk of EVD-specific death	0.21%	0.29%	0.33%	0.07%	0.56%	0.39%	0.39%	0.33%
**Vaccine effect**								
Vaccine effect and 95%CI†	100% (54.8 to 100)	100% (67 to 100)	92.3% (52.9 to 98.8)	100% (-3800 to 100)	100% (64.3 to 100)	100% (62.6 to 100)	92% (23.4 to 99.2)	88.8% (27.6 to 98.3)
p value‡	0.0257	0.0426	0.0588	1	0.0471	0.0102	0.0525	0.148

*For the secondary analysis, endpoints are confirmed EVD deaths with onset of symptoms at least 10 days post-randomisation.
** Randomised and non-randomised allocated to immediate vaccination are combined.
***Non-randomised immediate clusters are excluded from this analysis.
†From fitting a β-binomial distribution to the cluster-level numerators and denominators and using an inverted likelihood ratio test to identify the lower bound for vaccine efficacy (columns 1, 2, 5 and 6);
From Cox proportional hazards model (column 3, 7 and 8); From signed test (two-sided): probability of observing endpoints in control groups among treatment-control mismatched pairs and under the null hypothesis that the vaccine has no efficacy (column 4).
‡ From Fisher's exact test (two-sided), which is approximate for columns 1 and 2 . From sign test (two-sided): probability of observing endpoints in control groups among treatment-control mismatched pairs and under the null hypothesis that the vaccine has no efficacy (column 4).

Table S5: Distribution of confirmed Ebola virus disease cases among non-eligible CCCs

	Allocated to immediate vaccination	Allocated to delayed vaccination	Not randomised
Number of EVD cases (affected clusters)	9 (6)	13 (6)	8 (4)
Number of non eligible children	7/9 (77.8%)	11/13 (84.6%)	5/8 (62.5%)
**Confirmed EVD cases <10 days after randomisation**			
Number of EVD cases (affected clusters)	6 (5)	7 (5)	6 (3)
High-risk contact†	6/6 (100%)	7/7 (100%)	6/6 (100%)
**Confirmed EVD cases ≥10 days after randomisation**			
Number of EVD cases (affected clusters)	3 (2)	6 (3)	2 (2)
High-risk contact†	1/3 (33.3%)	6/6 (100%)	2/2 (100%)

† Contact information comes from the subject identification form for non-eligible subjects.

Table S6. Proportion of vaccinated participants (n = 5837) experiencing a solicited adverse event by time since vaccination.

	0 to 30 mins			31 mins to 3 days			4 to 14 days			Overall (0 to 14 days)		
Adverse events	Response rate (%)	N	%	Response rate (%)	N	%	Response rate (%)	N	%	Respons rate (%	N	%
Arthralgia	>99	3	0.1	95	854	15.4	90	80	1.5	88	919	17.9
Diarrhoea	>99	0	0	95	53	1	90	16	0.3	87	69	1.4
Fatigue	>99	5	0.1	95	1243	22.4	90	113	2.1	89	1312	25.4
Fever	>99	2	0	95	9	0.2	90	3	0.1	87	14	0.3
Headache	>99	41	0.7	95	1610	29	90	181	3.4	89	1739	33.3
Induration	>99	0	0	95	1	0	90	0	0	87	1	0
Injection Pain	>99	70	1.2	95	371	6.7	90	8	0.2	87	444	8.7
Muscle Pain	>99	7	0.1	95	879	15.8	90	56	1.1	88	928	18.1
Myalgia	>99	6	0.1	95	820	14.8	90	48	0.9	88	862	16.8
Vomiting	>99	0	0	95	22	0.4	90	4	0.1	87	25	0.5
Other AEs	>99	16	0.3	95	544	9.8	90	146	2.8	88	666	13
**Any AE†**	**>99**	**127**	**2.2**	**95**	**2972**	**53.4**	**90**	**468**	**8.9**	**92**	**3149**	**58.8**

† Response rate calculated as the proportion of participants who answered all AE fields or reported at least one AE.

Table S7: Severity of solicited adverse events in all vaccinated (n = 5837).

	Mild		Moderate		Severe		Unknown	
**Adverse events**	**n**	**%**	**n**	**%**	**n**	**%**	**n**	**%**
Arthralgia	835	89.1	95	10.1	7	0.7	0	0
Diarrhea	58	84.1	8	11.6	3	4.3	0	0
Fatigue	1147	84.3	193	14.2	21	1.5	0	0
Fever	0	0	0	0	0	0	14	100
Headache	1664	90.8	160	8.7	8	0.4	0	0
Induration	1	100	0	0	0	0	0	0
Injection Pain	402	89.5	30	6.7	17	3.8	0	0
Muscle Pain	785	83.3	148	15.7	9	1	0	0
Myalgia	728	83.3	136	15.6	10	1.1	0	0
Vomiting	19	73.1	4	15.4	3	11.5	0	0
Other AEs	672	95.2	19	2.7	5	0.7	10	1.4
**Total**	**6311**	**87.5**	**793**	**11**	**83**	**1.2**	**24**	**0.3**

Table S8: Reported Serious Adverse Events in all vaccinated (n = 5837).

SAE Tracking for Ebola Vaccine Ring Trial Guinea 07 April 2016

SAE #	Age at enrollment	Gender	Date of enrollment	Date of vaccination	Diagnosis	Causality assessment outcome	Date of onset of SAE	Date of resolution of SAE	Duration of SAE in days	SAE inclusion criteria	Outcome
1	50 years	Female	23.03.15	23.03.15	Road Traffic Accident (RTA)	Not Related	13.04.15	05:05:15	23	Life threatening	Recovered
2	52 years	Female	18.04.15	18.04.15	Ebola Virus Disease	Not Related	18.04.15	28.04.15	10	Life threatening	Death
3	61 years	Female	17.04.15	Not vaccinated	Ebola Virus Disease	Not Related	18.04.15	28.04.15	11	Life Threatening	Death
4	34 years	Male	03.04.15	Not vaccinated	Ebola Virus Disease	Not Related	10.04.15	17.04.15	8 days	Death	Death
5	27 years	Female	03.04.15	Not vaccinated	Ebola Virus Disease	Not Related	10.04.15	20.04.2015	11	Life threatening	Recovered
6	40 years	Male	17.04.15	17.04.15	Traumatism	Not Related	04.05.15	07.07.15	64	LifeThreatening	Recovered
7	35 years	Male	29.04.15	Not vaccinated	Ebola Virus Disease	Not Related	13.05.15	21.05.15	8	Life Threatening	Death
8	25 years	Male	01.05.15	01.05.15	Febrile Illness	Not Related	17.05.15	23.05.15	8	Hospitalization	Recovered
9	20 years	Male	26.04.15	Not vaccinated	Road Traffic Accident	Not Related	12.05.15	15.05.15	3	Life Threatening	Recovered
10	40 years	Male	30.04.15	22.05.15	Malaria	Not Related	23.05.15	25.05.15	3	Hospitalization	Recovered
11	29 years	Male	30.04.15	1.05.15	Febrile Illness	Not Related	25.05.15	31.05.15	7	Hospitalization	Recovered
12	35 years	Male	24.05.15	24.05.15	Reaction to Amoxicillin	Not Related	24.05.15	02.06.15	9	Hospitalization	Resolved
13	51 years	Male	06.05.15	6.05.15	RTA with multiple injuries	Not Related	21.05.15	30.06.15	41	Hospitalization	Recovered
14	27 years	Male	16.05.15	Not vaccinated	Ebola Virus Disease	Not Related	25.05.15	27.05.15	3	Death	Death
15	36 years	Male	19.05.15	19.05.15	RTA with Head Injury	Not Related	21.05.15	29.05.15	9	Hospitalization	Death
16	60 years	Male	23.03.15	23.05.15	Acute Appendicitis	Not Related	22.05.15	04.06.15	13	Hospitalization	Recovered
17	78 years	Male	15.05.15	Not vaccinated	Ebola Virus Disease	Not Related	27.05.15	31.05.15	4	Life threatening	Death
18	45 years	Male	15.05.15	Not vaccinated	Ebola Virus Disease	Not Related	01.06.15	15.06.15	15	Life threatening	Recovered
19	50 years	Female	16.05.15	Not vaccinated	Ebola Virus Disease	Not Related	15.05.15	21.05.15	6	Life threatening	Death
20	52 years	Female	15.05.15	Not vaccinated	Ebola Virus Disease	Not Related	26.05.115	17.06.15	23	Life Threatening	Death
21	60 years	Male	14.05.15	Not vaccinated	Ebola Virus Disease	Not Related	23.05.15	24.05.15	1	Life Threatening	Death
22	23 years	Male	13.05.15	03.06.15	Infection Syndrome	Not Related	04.06.15	09.06.15	5	Hospitalization	Resolved
23	51 years	Female	17.05.15	Not vaccinated	Ebola Virus Disease	Not Related	05.06.15	08.06.15	4	Life threatening	Death
24	18 years	Male	15.05.15	04.06.15	Ebola Virus Disease	Not Related	04.06.15	20.06.15	16	Hospitalization	Recovered
25	25 years	Male	30.05.15	30.05.15	Febrile Reaction	Related	30.05.15	02.06.15	3	Hospitalization	Resolved
26	21 years	Male	15.05.15	04.06.15	Ebola Virus Disease	Not Related	10.06.15	13.06.15	3	Life Threatening	Death
27	22 years	Female	15.05.15	04.06.15	Ebola Virus Disease	Not Related	10.06.15	15.06.15	6	Life Threatening	Death
28	50 years	Female	09.06.15	09.06.15	Ebola Virus Disease	Not Related	11.06.15	20.06.15	10	Life Threatening	Death
29	28 years	Male	22.05..15	22.05.15	Dysentery Syndrome	Not Related	28.05.15	03.06.15	6	Hospitalization	Recovered
30	70 years	Male	14.05.15	Not vaccinated	Ebola Virus Disease	Not Related	01.06.15	09.06.15	9	Life Threatening	Death
31	55 years	Female	29.04.15	Not vaccinated	Ebola Virus Disease	Not Related	30.04.15	09.05.15	10	Death	Death
32	62 years	Male	15.06.15	15.06.15	Ebola Virus Disease	Not Related	17.06.15	21.06.2015	5	Hospitalization	Death
33	29 years	Male	26.05.15	16.06.15	Ebola Virus Disease	Not Related	18.06.15	13.07.15	25	Hospitalization	Recoved
34	19 years	Male	14.06.15	14.06.15	Ebola Virus Disease	Not Related	15.06.15	02.07.15	17	Hospitalization	Resolved
35	45 years	Female	14.06.15	14.06.15	Ebola Virus Disease	Not Related	16.06.15	02.07.2015	17	Hospitalization	Resolved
36	33 years	Male	15.06.15	15.06.15	Ebola Virus Disease	Not Related	17.06.15	24.06.15	8	Hospitalization	Death
37	25 years	Male	15.04.15	15.04.15	Malaria	Not Related	14.06.15	18.06.15	5	Life Threatening	Recovered
38	70 years	Female	13.05.15	Not vaccinated	Ebola Virus Disease	Not Related	15.05.15	20.05.15	6	Hospitalization	Death
39	53 years	Male	16.06.15	16.06.15	Myocardial Infarction	Not Related	26.06.15	26.06.15	1	Death	Death
40	20 years	Male	15.05.15	03.06.15	Febrile Illness	Not Related	11.07.15	15.07.15	5	Hospitalization	Resolved
41	34 years	Male	12.07.15	12.07.15	Ebola Virus Disease	Not Related	13.07.15	20.07.15	8	Hospitalization	Death
42	65 years	Male	12.07.15	12.07.15	Ebola Virus Disease	Not Related	14.07.15	05.08.15	23	Hospitalization	Recovered
43	19 years	Female	12.07.15	12.07.15	Ebola Virus Disease	Not Related	13.07.15	08.08.15	26	Hospitalization	Recovered
44	60 years	Male	11.07.15	11.07.15	Febrile Syndrome	Not Related	13.07.15	18.07.15	5	Hospitalization	Recovered
45	18 years	Female	12.07.15	12.07.15	Ebola Virus Disease	Not Related	19.07.15	03.08.15	15	Hospitalization	Recovered
46	41 years	Female	27.06.15	27.06.15	HIV-TB co-infection	Not Related	31.07.15	01.08.15	2	Death	Death
47	19 years	Male	05.08.15	05.08.15	Ebola Virus Disease	Not Related	10.08.15	31.08.15	21	Hospitalization	Recovered
48	25 years	Male	02.08.15	02.08.15	Malaria	Not Related	10.08.15	13.08.15	4	Hospitalization	Recovered
49	26 years	Male	20.06.15	20.06.15	Blunt cranio-encephalic trauma	Not Related	09.08.15	28.08.15	20	Life threatening	Recovered
50	51 years	Female	05.08.15	05.08.15	Ebola Virus Disease	Not Related	12.08.15	17.08.15	5	Hospitalization	Death
51	35 years	Male	07.08.15	07.08.13	Ebola Virus Disease	Not Related	13.08.15	01.09.15	19	Hospitalization	Recovered
52	60 years	Female	06.08.15	06.08.15	Influenza- like Syndrome	Possibly Related	06.08.15	09.08.15	3	Hospitalization	Recovered
53	35 years	Female	07.08.15	07.08.15	Malaria	Not Related	13.08.15	17.08.15	4	Hospitalization	Recovered
54	30 years	Male	01.05.15	01.05.15	HIV-TB co-infection	Not Related	05.06.15	05.08.15	61	Hospitalization	Recovered
55	52 years	Male	20.08.15	20.08.15	Ebola Virus Disease	Not Related	20.08.15	20.08.15	0	Hospitalization	Death
56	18 years	Male	20.08.15	20.08.15	Malaria	Not Related	21.08.15	28.08.15	8	Hospitalization	Recovered
57	50 years	Female	11.07.15	11.07.15	Infected Wound on left hand	Not Related	21.08.115	09.09.15	19	Hospitalization	Recovered
58	20 years	Female	02.07.15	23.07.15	Spontaneous Abortion	Not Related	14.08.15	29.08.15	16	Hospitalization	Recovered
59	56 years	Male	19.08.15	19.08.15	Ebola Virus Disease	Not Related	27.08.15	03.09.15	7	Hospitalization	Death
60	75 years	Female	05.09.15	05.09.15	Ebola Virus Disease	Not Related	05.09.15	18.09.15	14	Hospitalization	Recovered
61	23 years	Female	04.09.15	04.09.15	Ebola Virus Disease	Not Related	06.09.15	26.09.15	22	Hospitalization	Recovered
62	36 years	Male	12.07.15	12.07.15	Ebola Virus Disease	Not Related	16.07.15	24.07.15	9	Hospitalization	Death
63	81 years	Male	19.06.15	19.06.15	Probable renal insufficiency	Not Related	12.09.15	12.09.15	1	Death	Death
64	70 years	Male	16.07.15	03.08.15	Anaphylaxis	Related	03.08.15	05.08.15	6	Life threatening	Recovered
65	56 years	Male	29.04.15	29.04.15	Erysipelas	Not Related	29.06.15	08.07.15	6	Hospitalization	Recovered
66	59 years	Male	21.09.15	21.09.15	Ebola Virus Disease	Not Related	21.09.15	03.10.15	13	Hospitalization	Death
67	22 years	Male	21.09.15	21.09.15	Ebola Virus Disease	Not Related	21.09.15	07.10.15	17	Hospitalization	Recovered
68	10 years	Male	16.10.15	16.10..15	Ebola Virus Disease	Not Related	22.10.15	19.11.15	28	Hospitalization	Recovered
69	70 years	Male	10.07.15	03.08.15	Intestinal obstruction from strangulated hernia	Not Related	26.10.15	26.10.15	0	Death	Death
70	31 years	Female	05.08.15	05.08.15	Fracture left ankle	Not Related	08.10.15	16.10.15	8	Hospitalization	Recovered
71	11 years	Female	15.10.15	15.10.15	Malaria	Not Related	30.10.15	03.11.15	5	Hospitalization	Recovered
72	40 years	Female	19.09.15	19.09.15	Malaria	Not Related	29.09.15	05.10.15	6	Hospitalization	Recovered
73	10 years	Male	28.10.15	28.10.15	Malaria	Not Related	30.10.15	02.11.15	3	Hospitalization	Recovered
74	21 years	Male	21.09.15	21.09.15	Acute Appendicitis	Not Related	16.10.15	25.10.15	9	Hospitalization	Recovered
75	35 years	Female	19.09.15	19.09.15	Malaria in Pregancy	Not Related	24.11.15	07.12.15	13	Hospitalization	Recovered
76	45 years	Male	07.07.15	28.07.15	Appendicitis	Not Related	18.10.15	26.10.15	8	Hospitalization	Recovered
77	10 years	Female	29.09.15	29.09.15	Umbilical hernia	Not Related	25.11.15	20.12.15	25	Hospitalization	Recovered
78	22 years	Female	21.09.15	21.09.15	Acute Appendicitis and urogenital infection	Not Related	01.12.15	15.12.15	15	Hospitalization	Recovered
79	56 years	Female	28.09.15	28.09.15	Gastroduodenal ulcer probably associated tumor	Not Related	01.12.15	05.12.15	5	Death	Death
80	40 years	Female	27.06.15	27.06.15	Spontaneous Abortion	Not Related	14.10.15	29.10.15	15	Hospitalization	Recovered

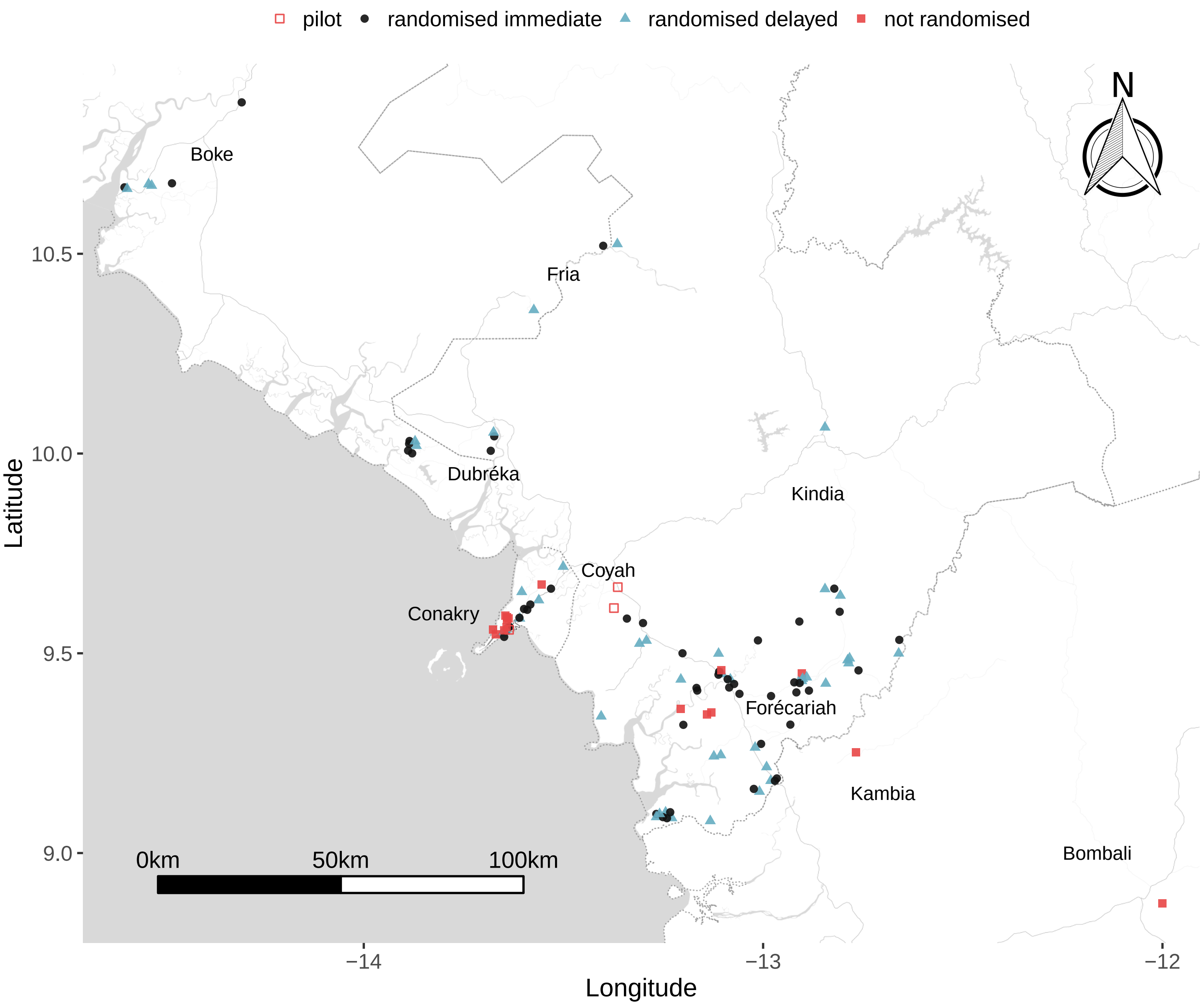

pilot
randomised immediate
randomised delayed
not randomised
N
Boke
Fria
Dubréka
Kindia
Coyah
Conakry
Forécariah
Kambia
Bombali
0km
50km
100km
10.5
10.0
9.5
9.0
−14
−13
−12
Latitude
Longitude

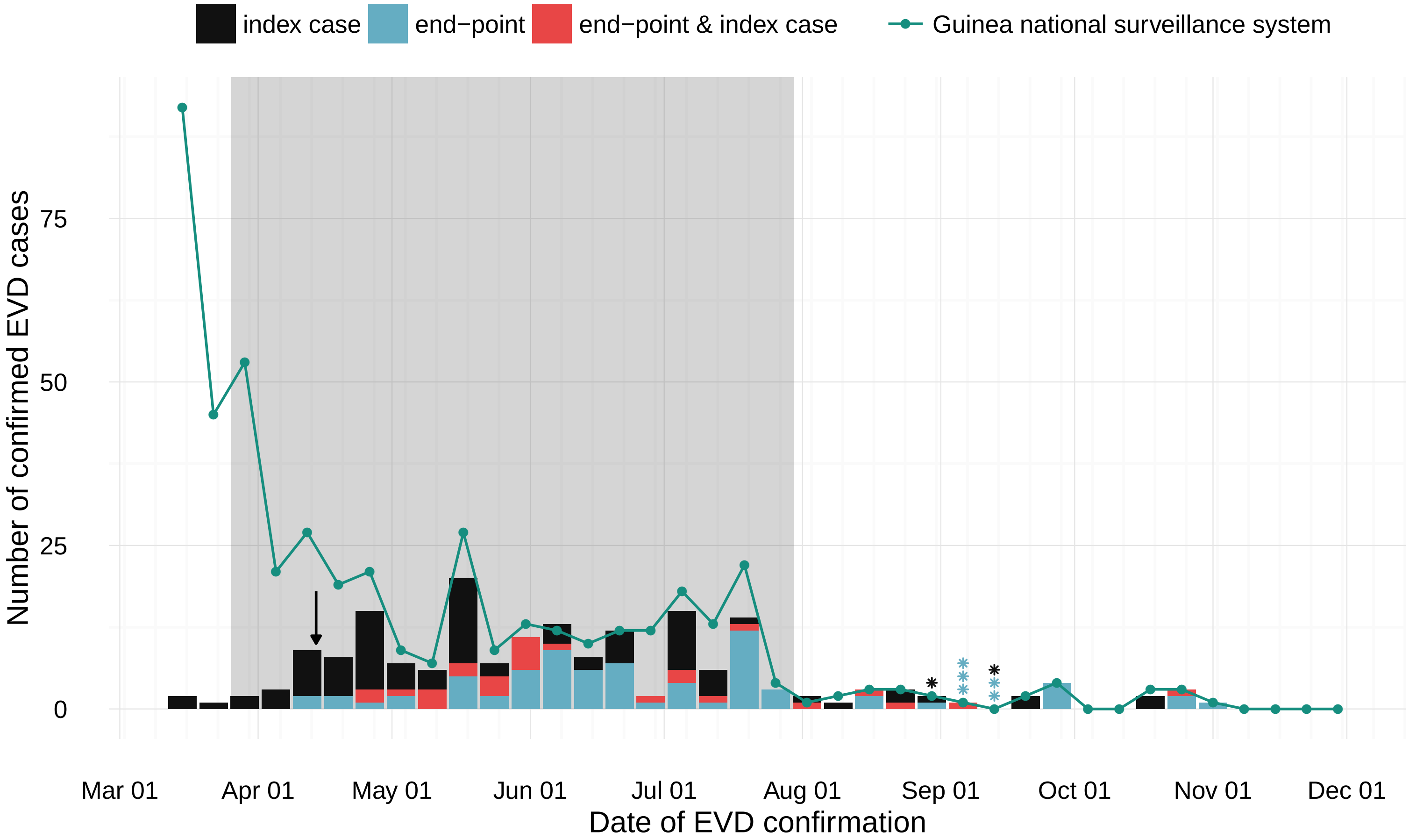
index case
end−point
end−point & index case
Guinea national surveillance system
Number of confirmed EVD cases
75
50
25
0
Mar 01
Apr 01
May 01
Jun 01
Jul 01
Aug 01
Sep 01
Oct 01
Nov 01
Dec 01
Date of EVD confirmation

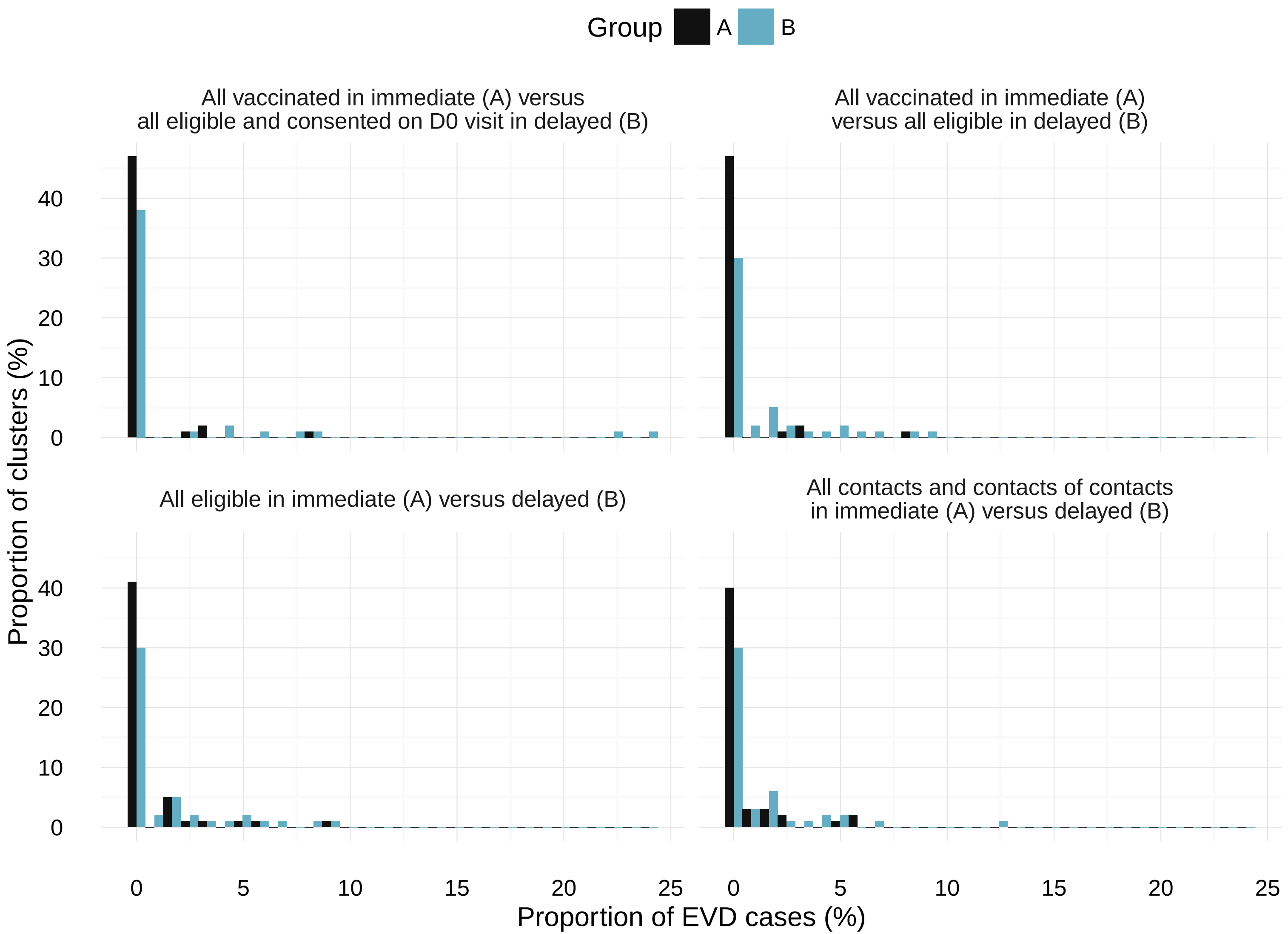
Group
A
B
All vaccinated in immediate (A) versus
all eligible and consented on D0 visit in delayed (B)
All vaccinated in immediate (A)
versus all eligible in delayed (B)
All eligible in immediate (A) versus delayed (B)
All contacts and contacts of contacts
in immediate (A) versus delayed (B)
Proportion of clusters (%)
0
10
20
30
40
0
5
10
15
20
25
Proportion of EVD cases (%)

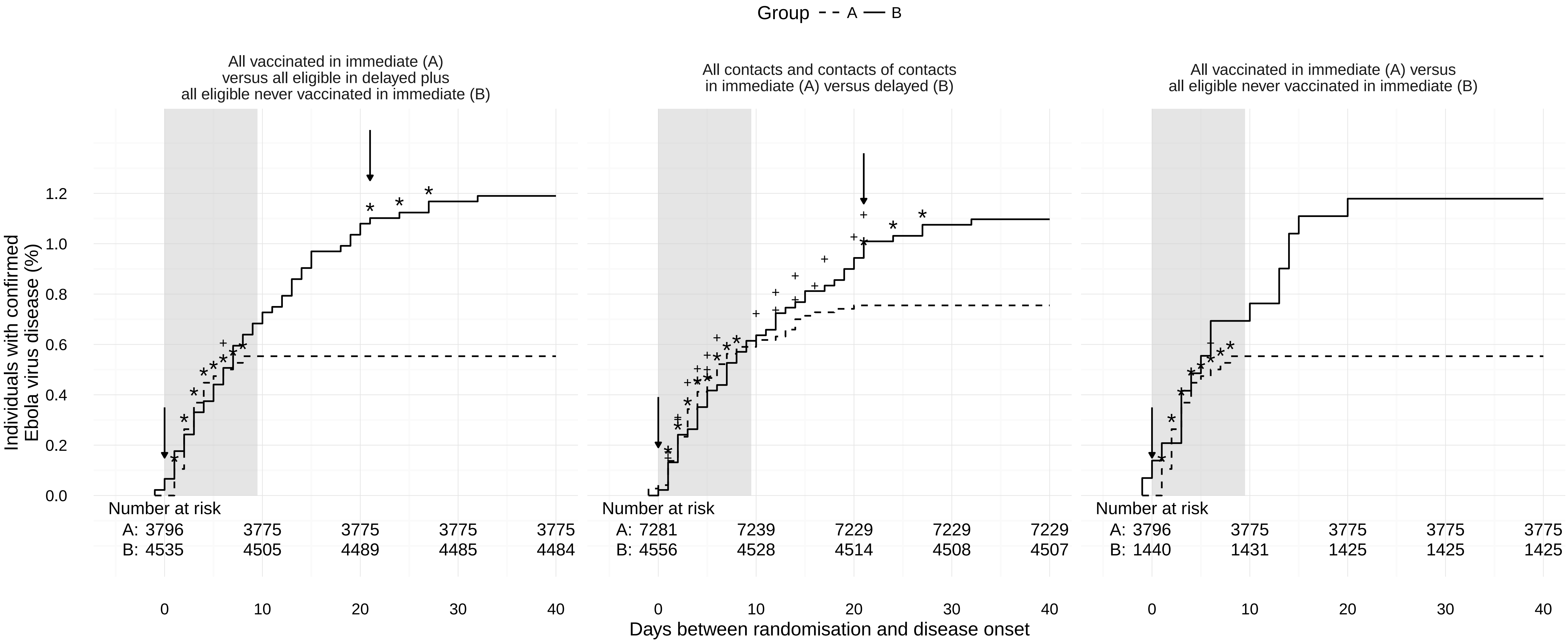

Group
A
B
All vaccinated in immediate (A) versus all eligible in delayed plus all eligible never vaccinated in immediate (B)
All contacts and contacts of contacts in immediate (A) versus delayed (B)
All vaccinated in immediate (A) versus all eligible never vaccinated in immediate (B)
Individuals with confirmed Ebola virus disease (%)
0.0
0.2
0.4
0.6
0.8
1.0
1.2
Number at risk
A: 3796 3775 3775 3775 3775
B: 4535 4505 4489 4485 4484
A: 7281 7239 7229 7229 7229
B: 4556 4528 4514 4508 4507
A: 3796 3775 3775 3775 3775
B: 1440 1431 1425 1425 1425
0
10
20
30
40
Days between randomisation and disease onset
